# Supplementary material for: Age-Dependent Alterations in Semen Parameters and Human Sperm MicroRNA Profile
Source: Biomedicines. 2023 Oct 28;11(11):2923. doi: 10.3390/biomedicines11112923 (PMC10669352; doi:10.3390/biomedicines11112923)
Supplement: Supplementary file 1 [file biomedicines-11-02923-s001.zip › Figure S1.pdf]

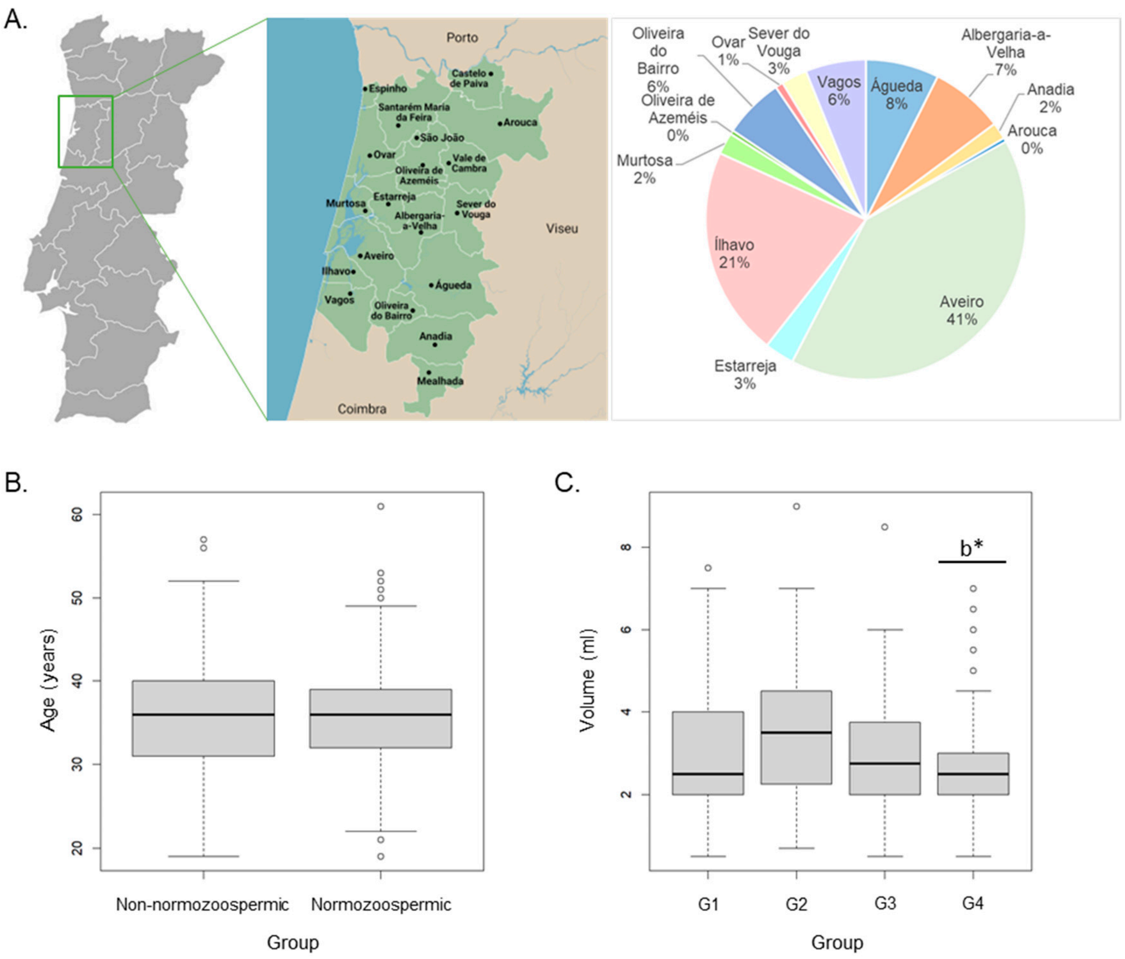

Figure S1 – Geographical distribution of the 333 participants integrating this study (A). The mean age of the participants with altered semen parameters (non-normozoospermic patients) was not significantly different from the participants with normal conventional semen parameters (normozoospermic patients) (B). Men with advanced paternal age (> 40 years old) presented significantly lower semen volume than men aged. b* represent significant differences from 31 - 35 years (p-value < 0.05).
